# Editorial: Work and Brain Health Across the Lifespan

**DOI:** 10.3389/fnhum.2021.741582

**Published:** 2021-08-13

**Authors:** Agnieszka Z. Burzynska

**Affiliations:** ^1^Department of Human Development and Family Studies, Colorado State University, Fort Collins, CO, United States; ^2^Department of Molecular, Cellular and Integrative Neurosciences, Colorado State University, Fort Collins, CO, United States

**Keywords:** brain, work, occupation, physical activity, stress, aging, occupational neuroscience, pandemic (COVID19)

This Research Topic aimed to contribute empirical evidence to the new field of study that we called “occupational neuroscience” (Burzynska et al., 2019), which aims to understand the effects of occupational factors on adult brain health. Here, I will discuss the work presented in this Research Topic in the context of the “Brain aging: Occupational Stimulation and Stress” (BOSS) model we proposed earlier. The BOSS model assumes that work environment can accelerate age-related decline in brain health through exposure to occupational stress, sedentariness, and environmental hazards, or protect the aging mind and brain through cognitive stimulation, physical activity, and environmental enrichment (Burzynska et al., 2019). One of the key assumptions of the BOSS model is that there is a long-term interplay between these protective and risk influences.

In the first study, Bergman et al. reported the effects of a 13-month physical activity intervention on measures of cognitive and brain health in office workers. The unique aspects of this clinical trial include: (a) targeting physical activity at workplace (as opposed to interventions increasing exercise during leisure time), (b) unstructured and, therefore, more ecologically-valid workplace physical activity, encouraged by treadmill workstation availability and email reminders, and (c) involvement of overweight-to-obese, middle-aged participants (40–67 years) performing sedentary office work, namely, a population at potentially increased risk of cognitive and brain health decline, yet possibly decades before these declines manifest as cognitive impairment. Although there were no time × group effects on brain or cognitive measures, the authors report a positive association between *change* in walking time and light physical activity on work days and a *change* in hippocampal volume, and a negative cross-sectional association between percentage time spent sitting and hippocampal volume, both associations being driven by workers older than 51 (Bergman et al.).

In the second study, we related retrospective measures of subjective midlife occupational complexity, physical stress, and psychological stress to hippocampal volume in older age (Burzynska et al.). We found that only perceived occupational physical stress, comprising physical demands, and work conditions, was associated with smaller hippocampal volume and poorer memory performance. As this association was independent of job title (and therefore, of objective physical demand status of the job) as well as leisure physical activity, our results suggest that perceived (and potentially unwelcome) physical demands at work can represent an important stressor and that leisure PA may have largely independent effects on brain and cognitive health (Burzynska et al.).

In the third study, Habeck et al. investigated how characteristics of the longest reported occupation relate to structural brain health in adults 40–80 years old. The unique aspect of this study is the fine-grained analysis of 246 occupational factors across the dimensions of work values, interests, knowledge, abilities, work activities, work styles, skills, and work context, distinct from the majority of existing studies using broad self-reported job characteristics or crude job title classifications. Furthermore, the multimodal brain health score reflected a combination of global cortical thickness, white matter integrity and white matter hyperintensity volume. The analyses revealed that work-related rigorous problem-solving, numerical ability, information processing, leadership, and responsibility positively correlated with the global brain health score, beyond the effects of age, education, IQ, and gender (Habeck et al., 2019).

Together, the results of these studies support and extend the BOSS model (Figure 1). First, occupational stimulation as the anti-aging factor for brain health has been extended to objective job classifications related to rigorous problem-solving, leadership, responsibility, information processing (Habeck et al., 2019), but not to perceived cognitive job complexity (Burzynska et al.). Second, work-related physical activity and sedentariness have been added as a new dimension of the BOSS model. Specifically, work-place sedentariness (Bergman et al.) and perceived physical demands (Burzynska et al.) were identified as pro-aging stressors, whereas replacing or interrupting work-related sedentariness with light physical activity may slow down age-related decline in hippocampal volume (Bergman et al.) (Figure 1). Importantly, with regard to theories of neurocognitive aging, the three studies presented here support the brain maintenance theory (Nyberg, 2017), where cognitive stimulation and physical activity minimize and physical stressors amplify age-related neural decline [as opposed to cognitive reserve model, in which occupational enrichment would show negative association with brain health (Stern et al., 2018)].

**Figure 1:**
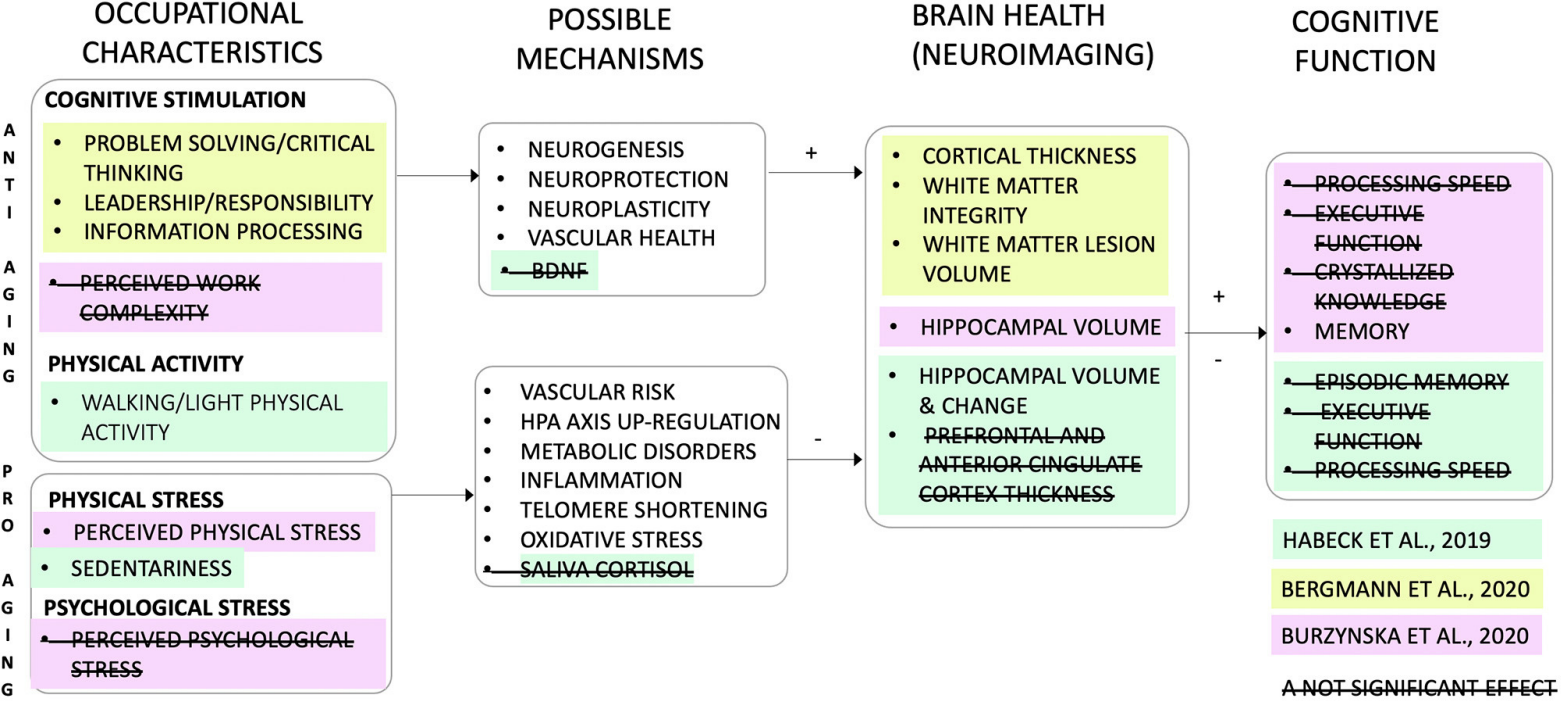
The BOSS model (Brain aging: Occupational Stimulation and Stress; Burzynska et al., 2019): revised and extended. The model assumes two parallel and possibly opposing influences of occupations on brain health: the “anti-aging” (or neuroprotective) and “pro-aging” (or accelerating aging). The studies in the Research Topic “Work and Brain Health Across the Lifespan” add data supporting the anti-aging role of light physical activity and cognitive and social demands at work, and pro-aging effects of work-related sedentariness and perceived physical demands. One study included possible physiological mechanisms (i.e., peripheral blood levels of brain-derived neurotrophic factor, BDNF, and saliva cortisol), but the mediation results were not significant (Bergman et al.). The only significant association of work characteristics with cognition was the negative association between perceived physical demands and memory (Burzynska et al.). Colors indicate the three studies in the Research Topic. Strikethrough indicate non-significant findings. Text in white indicate mechanisms that were not studied. HPA, hypothalamic–pituitary–adrenal axis.

One of the future directions for research on “occupational neuroscience,” evident from Figure 1, is the need to study physiological mechanisms linking occupational exposure with brain health. These should include, for example, blood markers of inflammation, oxidative stress, metabolic, and neuroendocrine health, as well as measures of sleep quality and cardiovascular health. Second, longitudinal studies are urgently needed to test neural and physiological mechanisms or mediators of work-cognition relationships. A mediation cannot establish causation, but given that randomized clinical trials involving substantial occupational changes are not feasible, absence of a mediation can help disconfirm a hypothesis on causal relations (Salthouse, 2011).

Finally, as this editorial is written in June 2021, I feel compelled to point out possible effects of the pandemic-related disruptions to our occupational lives on the adult brain health. The pandemic forced 24% of the total U.S. workforce to work remotely (U.S. Bureau of Labor Statistics, 2020, Table 2). Remote work reduced work-related physical activity and significantly increased sitting and screen time (McDowell et al., 2020). The work-life boundary got blurred, resulting in the average 3-h work time increase/workday in the U.S. since the COVID pandemic began (Williams, 2020). Conversely, health care workers and first responders were exposed to long-term psychological and physical stress, whereas others had to deal with employment uncertainty. The differences in work-family conflict experienced by women and men during the pandemic (Ganster, submitted) as well as differences in the return to in-person work between companies provide unique opportunities for studying the effects of the gender inequality and remote work on adult brain and cognitive health, respectively. Taken together, future research, possibly using the BOSS framework, is needed to determine whether changes in occupational life during the pandemic will have any long-lasting effects of on brain and cognitive aging of the current workforce population.

## Author Contributions

The author confirms being the sole contributor of this work and has approved it for publication.

## Conflict of Interest

The author declares that the research was conducted in the absence of any commercial or financial relationships that could be construed as a potential conflict of interest.

## Publisher's Note

All claims expressed in this article are solely those of the authors and do not necessarily represent those of their affiliated organizations, or those of the publisher, the editors and the reviewers. Any product that may be evaluated in this article, or claim that may be made by its manufacturer, is not guaranteed or endorsed by the publisher.
